# Training health professionals to recruit into challenging randomized controlled trials improved confidence: the development of the QuinteT randomized controlled trial recruitment training intervention

**DOI:** 10.1016/j.jclinepi.2017.11.015

**Published:** 2018-03

**Authors:** Nicola Mills, Daisy Gaunt, Jane M. Blazeby, Daisy Elliott, Samantha Husbands, Peter Holding, Leila Rooshenas, Marcus Jepson, Bridget Young, Peter Bower, Catrin Tudur Smith, Carrol Gamble, Jenny L. Donovan

**Affiliations:** a MRC ConDuCT-II Hub for Trials Methodology Research, Population Health Sciences, Bristol Medical School, University of Bristol, Canynge Hall, 39 Whatley Road, Bristol BS8 2PS, UK; b Nuffield Department of Surgical Sciences, University of Oxford, John Radcliffe Hospital, Headington, Oxford OX3 9DU, UK; c MRC North West Hub for Trials Methodology Research, Institute of Psychology Health and Society, University of Liverpool, Block B, Waterhouse Building, Brownlow Street, Liverpool L69 3GL, UK; d MRC North West Hub for Trials Methodology Research, Centre for Primary Care, University of Manchester, Williamson Building, Manchester M13 9PL, UK; e MRC North West Hub for Trials Methodology Research, Institute of Translational Medicine, University of Liverpool, Block F Waterhouse Building, 1-5 Brownlow Street, Liverpool L69 3GL, UK; f NIHR Collaboration for Leadership in Applied Health Research and Care (CLAHRC), University Hospitals Bristol NHS Foundation Trust, Whitefriars, Lewins Mead, Bristol, BS1 2NT, UK

**Keywords:** Equipoise, Patient treatment preference, Professional education, Randomized controlled trial, Recruitment to randomized controlled trials, Training health professionals

## Abstract

**Objectives:**

The objective of this study was to describe and evaluate a training intervention for recruiting patients to randomized controlled trials (RCTs), particularly for those anticipated to be difficult for recruitment.

**Study Design and Setting:**

One of three training workshops was offered to surgeons and one to research nurses. Self-confidence in recruitment was measured through questionnaires before and up to 3 months after training; perceived impact of training on practice was assessed after. Data were analyzed using two-sample *t*-tests and supplemented with findings from the content analysis of free-text comments.

**Results:**

Sixty-seven surgeons and 32 nurses attended. Self-confidence scores for all 10 questions increased after training [range of mean scores before 5.1–6.9 and after 6.9–8.2 (scale 0–10, all 95% confidence intervals are above 0 and all *P*-values <0.05)]. Awareness of hidden challenges of recruitment following training was high—surgeons' mean score 8.8 [standard deviation (SD), 1.2] and nurses' 8.4 (SD, 1.3) (scale 0–10); 50% (19/38) of surgeons and 40% (10/25) of nurses reported on a 4-point Likert scale that training had made “a lot” of difference to their RCT discussions. Analysis of free text revealed this was mostly in relation to how to convey equipoise, explain randomization, and manage treatment preferences.

**Conclusion:**

Surgeons and research nurses reported increased self-confidence in discussing RCTs with patients, a raised awareness of hidden challenges and a positive impact on recruitment practice following QuinteT RCT Recruitment Training. Training will be made more available and evaluated in relation to recruitment rates and informed consent.

**What is new?**

**Key findings**
• A training workshop for health professionals that focuses on the emotional and intellectual challenges of recruiting patients to randomized controlled trials (RCTs) looks promising in increasing confidence with recruitment, raising awareness of hidden challenges, and impacting positively on self-assessed recruitment practice.

**What this adds to what was known?**
• A recent systematic review of training interventions for RCT recruiters concluded more work was required to address unmet training needs.
• We built on previous training interventions by delivering training that raised awareness of hidden emotional and intellectual challenges of recruiting to RCTs, showing positive impacts on self-confidence and self-reported improvements in conveying clinical equipoise, explaining randomization, and managing patients' treatment preferences.

**What is the implication and what should change now?**
• Recruiters can be trained to be aware of and address hidden discomfort with recruiting patients to RCTs.
• The training intervention will continue to be developed, made more available, and be evaluated in relation to informed consent and impact on recruitment rates.

## 1 Introduction

Randomized controlled trials (RCTs) are regarded as the most rigorous study design to evaluate the effectiveness of health care interventions, but recruitment to them can be challenging. Less than 50% of randomized trials have been estimated to meet their recruitment targets [1]. Low rates of recruitment have implications not only for the internal and external validity of the trial but also financial and ethical consequences, delaying the introduction of effective treatments. RCTs involving surgical interventions have particular challenges, which have been linked to surgeons' lack of familiarity with RCT methodology and appreciation of the need for them [2]. Consequently, up to one in three surgical RCTs close prematurely or findings from completed ones are not published [3], [4]. Despite research over a number of years to understand the barriers to trial recruitment [5], [6], [7], [8] and interventions designed to address them [1], [9], recruitment difficulties are poorly understood and still persist.

Clinician-related factors have been cited as one of the main reasons for low rates of recruitment in RCTs across different care settings [10], [11]. Even though nurses have taken on an increasing role in RCT recruitment in the UK in recent years, this is often without much formal training beyond the required Good Clinical Practice training that offers broad ethical guidance on clinical trial conduct. A systematic review of the recruitment activity of clinicians across a variety of RCTs highlighted the need for training for recruiters [12]. The authors concluded that understanding and communicating RCT methods were a priority for future recruiter directed interventions to improve recruitment. Findings from a survey and workshop of UK Clinical Research Collaboration registered Clinical Trials Unit directors confirmed these findings [13]. They considered methods for improving recruitment, and in particular training for site staff, as the highest priority for RCT methodology research. Despite these recommendations, robust research into the development and evaluation of recruiter training programs designed to address recruiter needs is limited. A recent systematic review of training interventions for recruiters to RCTs found evidence of improvements in recruiters' self-confidence in communicating with patients about RCTs following training [14]. However, recruiters still struggled with the amount, clarity, and completeness of information to provide to patients and had difficulty with explaining key RCT concepts such as randomization. The authors concluded that more work is required to address these identified training needs.

Previous RCT recruiter training programs have tended to focus on communication of key issues (what to convey and how to convey it) [15], [16], [17], [18], [19], [20], [21], [22], [23], but the issues are not always that obvious. The QuinteT (Qualitative research integrated within Trials) research team has developed a recruitment intervention (QuinteT Recruitment Intervention [QRI]) which has been embedded in RCTs to understand and address recruitment difficulties [24]. This has advanced our understanding of the intricacies of the recruitment process revealing difficulties beyond communication that relate to emotional and intellectual challenges with approaching and recruiting patients [25], [26]. The research confirms conclusions from previous studies that RCT recruiters could benefit from support, especially in relation to emotional aspects of recruitment work [27]. It also highlights the need for training programs to raise awareness of how recruiters' views and discomfort can contribute to recruitment difficulties and for training to be tailored to meet the differing needs of doctors and nurses. Findings from this, and related research [28], [29], have been developed into training materials to improve the practice of frontline and future RCT recruiters. This paper describes the QuinteT RCT Recruitment Training and evaluates its impact on surgeons' and research nurses' perceived levels of:

(a) self-confidence in recruiting patients to surgical RCTs
(b) awareness of key challenges to effective recruitment
(c) self-assessed recruitment practice.

## 2 Methods

### 2.1 Participants

Surgeons and research nurses who were actively recruiting to an RCT that involved surgery, or those who planned to recruit to one within the next 12 months, were invited to attend one of four training workshops relevant to their profession (three dates were offered to surgeons and one to nurses). RCTs that were known or anticipated to have recruitment difficulties were primarily targeted. The chief investigator of these trials (or in a few cases the trial manager) invited and encouraged recruiters to attend. Workshops were also advertised through the Royal College of Surgeons, the Medical Research Council's Network of Hubs for Trial Methodology Research, and the University of Bristol's Surgical Trials Centre.

### 2.2 Workshop development and delivery

Three 1-day workshops lasting 5 hours each were held between March and May 2015 and one in May 2016. The workshops were broadly similar, comprising interactive presentations, group exercises, and discussion based around key recruiter challenges for the different health professionals. The aims of the training were to: (1) share skills and evidence-based knowledge, (2) promote awareness and tackling of key recruitment challenges, and (3) enhance self-confidence in recruiting patients to RCTs.

#### 2.2.1 Content

**Fig. 1:**
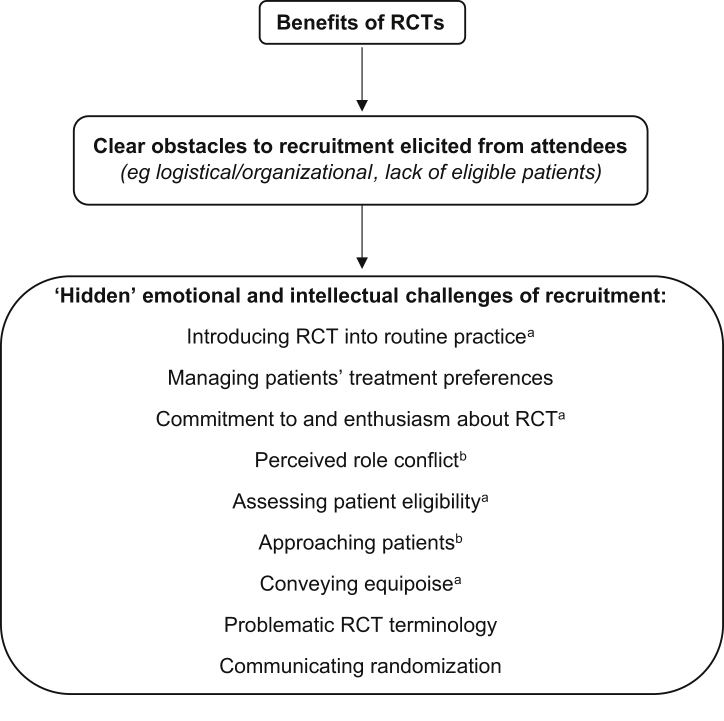
Content of the workshops. ^a^Covered in greater depth with surgeons; ^b^Covered in greater depth with research nurses. RCT, randomized controlled trial.

Fig. 1 shows the content of the workshops. The material was empirically based, addressing the clear obstacles and hidden challenges of recruitment identified from a synthesis of QRI studies embedded within six pragmatic RCTs [25], [26] and supplemented with findings from related studies [28], [29], across a range of clinical settings. The same topics were covered in each workshop but to varying degrees depending on whether it was delivered to nurses or surgeons (Fig. 1). Verbatim quotes from audio recordings of RCT recruitment discussions were used extensively to demonstrate what other recruiters had said in relation to the key topics and to show the impact that this had on patients' decision-making. Discussion of issues typically followed in small or large groups. Participants were given a one-page summary sheet of key learning points at the end of training. (See web-based Supplemental Information/Appendix at www.jclinepi.com for the workshop program and key learning points).

#### 2.2.2 Delivery

Method of delivery was determined by the organizers' past experiences of recruiter training in individual RCTs [24] and findings from other studies on recruiter training [14]. The training was delivered through a mixture of informal slide presentations that encouraged interaction; role-play, one-to-one, and small group discussion with other course participants; and whole group discussion. With the exception of the first workshop (which was given primarily by an academic professor), workshops were delivered by a mixture of academic researchers, an academic surgeon, a research nurse, and a statistician/trialist. All had an extensive understanding of recruitment difficulties and previous recruiter training experience, and in two cases, direct experience of RCT recruitment.

Although the content and delivery of the surgeon workshops was essentially the same, we took into account feedback from attendees. The value of the interactive discussions was reported so more time was devoted to this in subsequent workshops. As we were continuing our research into understanding recruitment barriers in specific RCTs alongside this training, we also updated a few quotes highlighting discomfort with recruitment in later workshops.

### 2.3 Data collection

Questionnaires were given to workshop attendees at three time points: immediately prior to, immediately after, and at 1–3 months posttraining. Table 1 shows the data collected at the different time points. Before and immediately after training questionnaires were given to attendees on the day of the workshop. Follow-up questionnaires were emailed for electronic completion at 1, 2, and 3 months' posttraining. Attendees were asked to complete the survey at 1 or 2 months after the workshop if they had since engaged with patients about recruitment to an RCT or to complete the survey at 3 months follow-up regardless of whether they had or had not discussed recruitment since training.

**Table 1:** Questionnaire data

Pretraining Basic demographic data and experience of RCT recruitment Self-confidence on 10 aspects of recruiting patients to surgical RCTs on a scale from 0 (no confidence) to 10 (very confident) Immediately after training Usefulness of training on a scale from 0 (low) to 10 (high) in terms of: Amount learnt Awareness now of recruitment challenges Confidence now in discussing recruitment with patients Difference in the workshop will make to future discussions 1–3 mo posttraining Self-confidence on 10 aspects of recruiting patients to surgical trials (as pretraining questionnaire) Usefulness of training in discussions with patients (scale from 0 [low] to 10 [high]) Difference the training has made to discussions with patients (4-point Likert scale)

*Abbreviation:* RCT, randomized controlled trial.

The self-confidence questions were adapted from those developed by Jenkins et al. [18] to evaluate a training intervention for those recruiting to cancer RCTs, making them relevant for surgical RCTs (the specific questions can be seen in Table 3).

**Table 2:** Participants' characteristics and experience with recruitment to RCTs prior to training

Characteristics	Surgeons, *N* = 65,^a^^,^^b^*n* (%)	Research nurses, *N* = 32, *n* (%)	All participants, *N* = 97,^a^*n* (%)
Gender^c^			
Male	45 (73)	4 (13)	49 (53)
Female	17 (27)	27 (87)	44 (47)
Age (yr)^d^			
<20	0 (0)	0 (0)	0 (0)
20–29	8 (13)	2 (6)	10 (10)
30–39	30 (47)	5 (16)	35 (36)
40–49	24 (38)	14 (44)	38 (40)
50–59	1 (2)	10 (31)	11 (11)
60+	1 (2)	1 (3)	2 (2)
Number of years recruiting to RCTs^e^			
0	13 (20)	1 (3)	14 (15)
<1	19 (30)	12 (38)	31 (32)
1–2	16 (25)	6 (19)	22 (23)
3–5	6 (9)	6 (19)	12 (13)
>5	10 (16)	7 (22)	17 (18)

*Abbreviation:* RCT, randomized controlled trial.

a Two surgeons did not complete questionnaire.

b Thirty-five consultants, 10 registrars, and 20 surgical trainees.

c Missing gender data: 3 surgeons and 1 research nurse.

d Missing age data: 1 surgeon.

e Missing experience data: 1 surgeon.

**Table 3:** Self-confidence in discussing and recruiting patients to RCTs before and after training

Questions on confidence	Mean self-confidence scores^a^							
	Surgeons				Research nurses			
	Before training *n* = 65^b^	1–3 mo after training *n* = 51^b^	Score change^c^ (95% CI)	*P*-value	Before training *n* = 32^b^	1–3 mo after training *n* = 26^b^	Score change^c^ (95% CI)	*P*-value
How confident are you about discussing RCTs involving surgery with patients?	6.5	7.4 (*n* = 48)	↑0.9 (0.2, 1.7)	0.0122	6.4	8.2	↑1.8 (0.8, 2.7)	0.0004
How easy do you find describing randomization?	6.5	7.4 (*n* = 47)	↑0.9 (0.4, 1.5)	0.0019	6.9	7.8	↑0.9 (0.0, 1.7)	0.0388
How comfortable are you with explaining uncertainty about the best treatment to patients (i.e., clinical equipoise)?	6.1	7.6 (*n* = 48)	↑1.5 (0.8, 2.1)	<0.0001	6.2	7.9 (*n* = 25)	↑1.7 (1.0, 2.4)	<0.0001
How confident are you about providing complex information about RCTs to highly intelligent patients?	6.2	7.9	↑1.7 (1.1, 2.4)	<0.0001	5.8	7.5	↑1.7 (1.0, 2.5)	<0.0001
How confident are you about providing complex information about RCTs to patients with limited capacity to understand?	5.4	6.9 (*n* = 47)	↑1.5 (0.8, 2.1)	0.0001	5.8	7.3	↑1.5 (0.7, 2.5)	0.0010
How comfortable are you with entering patients into RCTs that compare surgery with no surgery or some other option?^d^	5.8 (*n* = 47)	7.6 (*n* = 31)	↑1.8 (0.8, 2.9)	0.0004	5.7	7.5 (*n* = 23)	↑1.8 (0.8, 2.8)	0.0009
How confident are you in dealing with the “internet guru” patient?	5.7	7.4 (*n* = 47)	↑1.7 (1.0, 2.3)	<0.0001	5.9 (*n* = 31)	7.7	↑1.8 (1.2, 2.5)	<0.0001
How confident are you in obtaining authentic informed consent for randomization from patients who have a deferential attitude toward you?	5.4	6.9 (*n* = 45)	↑1.5 (0.9, 2.1)	<0.0001	5.4	7.7 (*n* = 25)	↑2.3 (1.4, 3.1)	<0.0001
How confident are you when discussing RCTs with patients who are mistrustful and suspicious about trials and experiments in medicine?	5.1	7.1 (*n* = 46)	↑2.0 (1.4, 2.6)	<0.0001	5.8	7.5	↑1.7 (0.9, 2.6)	0.0001
How confident are you when dealing with patients who object to being randomized and who wish to choose their treatment?	5.5 (*n* = 64)	7.3 (*n* = 47)	↑1.8 (1.0, 2.5)	<0.0001	6.0	7.9	↑1.9 (1.1, 2.8)	<0.0001

*Abbreviation:* RCT, randomized controlled trial.

a On a scale from 0 (no confidence) to 10 (very confident).

b Number who responded (*n* given for individual question if different to this).

c Arrow denotes direction of change (increase or decrease).

d Administrative error means surgeons' responses to this question from the third workshop have not been included.

Attendees were encouraged to comment on their responses if they wished. Questionnaires were anonymous, given the small number of participants and potential for identification, with the exception of those used in the final workshop a year later. Here, participants were allocated a study number to enable identification and contact of nonresponders.

### 2.4 Data analysis

Two-sample *t*-tests (assuming equal variance), means, and standard deviations (SDs) were calculated in Stata v14.1. Although participants in the final surgeon workshop were allocated a study ID, data were analyzed as they were for the previous workshops, that is, comparison of group mean scores before and after training. We initially analyzed self-confidence data from the three surgeon workshops individually. Although there were some differences across groups in their baseline scores which may indicate a group effect, the score changes after training were consistently in the same direction and the confidence intervals (CIs) overlap each other. This gave us confidence in the robustness of the findings so we have presented collated findings from all three surgeon workshops for more precise estimates. Free text was analyzed to illustrate and elaborate on the numerical findings using content analysis to systematically categorize and describe the data [30]. Comments were read several times to get an overall sense of what was said and to create general categories which were then applied to the text. These categories were then further scrutinized to create subcategories, bearing in mind the question from which the comments came from. In the presented quotes, “RN” refers to research nurse and S1-3 refers to surgeon and the workshop that they attended.

## 3 Results

### 3.1 Characteristics of workshop participants

Ninety-nine participants (67 surgeons and 32 research nurses) attended one of four workshops relevant to their profession. All participants (with two exceptions) completed a questionnaire prior to training, all except one completed one immediately after training, and 72% (48/67) of surgeons and 81% (26/32) of nurses completed a questionnaire at 1- to 3-month follow-up. Around two-thirds of participants provided at least one free-text comment at one time point. Surgeons were involved with (or in a few cases soon to be involved with) over 40 different RCTs evaluating a surgical intervention, 35 for the nurses. The majority of attendees were actively recruiting to at least one RCT that the University of Bristol was involved with. Key recruiters from these trials were present. Those who attended the surgeon workshops were predominately male (73%) aged 30–39, and those at the nurse workshop were primarily female (87%) aged 40–49 years (Table 2). Just over half of the surgeons were consultants. The nurse attendees were more likely to have had some experience of recruiting patients into RCTs than the surgeons (97% of nurses had previous recruitment experience compared with 80% of surgeons), with a fifth of nurses having over 5-year experience (compared with 16% of surgeons) (Table 2).

### 3.2 Impact of training on self-confidence

#### 3.2.1 Self-confidence prior to training

Levels of self-confidence in discussing various aspects of RCT recruitment before training were similar between surgeons and nurses and tended to hover just over midway between not at all confident (0) and very confident (10) (range for surgeons 5.1–6.5; range for nurses 5.4–6.9) (Table 3). Surgeons were least confident when discussing RCTs with patients who are mistrustful and suspicious about RCTs and experiments in medicine, and nurses lacked confidence in obtaining authentic informed consent for randomization from patients who have a deferential attitude toward them.

Free-text comments noted by participants prior to training offered insight into reasons for midlevel confidence scores (Table 4, Box A). Both nurses and surgeons with minimal experience of recruitment expressed unease discussing RCTs with patients if it compared surgery with no surgery/treatment. Also, surgeons in particular were concerned with ensuring truly informed consent and dealing with patients' preconceived beliefs and fears about medical research. Some of the more experienced surgeons reported struggling with recruitment when they were not in equipoise (Table 4, Box A).

**Table 4:** Illustrative quotes from free-text responses^a^

Box A: Self-confidence prior to training My concern is approaching patients for recruitment and randomization when it's explained to them that randomization may be to no treatment at all *(RN, <1 year recruiting experience)* [Concerned with] being sure that people understand the trial I am explaining fully and the uncertainties they have about what placebo means/the fear of “not getting the best treatment” *(S3, 1–2 years recruiting experience)* I am not always sure the patients fully understand what they are getting into, especially the “nice” “trusting” ones *(S1, 1–2 years recruitment experience)* [Difficulties] if [I'm] not convinced of equipoise *(S1, >5 years recruiting experience)* Box B: Self-confidence after training It has given me more confidence discussing RCTs and randomization and also a structure to organize my discussion with patients *(S3)* I feel better at trying to balance interventions and explain that no one knows which is best, whilst maintaining their confidence that they will receive the best possible treatment for them (i.e., their care isn't compromised by being in a trial) *(RN)* I feel more confident in general in consenting patients and trying to explore reasons they might not want to take part instead of just accepting a no and ensuring they have all the information needed to make a decision *(S3)* Box C: Impact of training on raising awareness of recruitment challenges 1. Awareness of challenges post training Good thoughtful discussions and will make me consider my own practice. *(S1)* Good list of things to be aware of *(S2)* I am now aware of my lack of skills and how to overcome difficulties *(S2)* Very interesting day, with lots of thought provoking information *(RN)* 2. Value of discussing challenges Very useful techniques covered to increase recruitment for trials - especially time and order in which treatment options are discussed with patients and emphasis on exploring patients' ideas, concerns and expectations *(S3)* Learned importance of positive language about all treatment arms *(S2)* Know which words/terminology to avoid/use there and then *(S3)* Great to highlight importance of exploring patient's perspectives, and not consider this to be any kind of pressure to participate, but an important part of informed consent *(S3)* The part on equipoise was very useful as sometimes this is compromised. It was interesting to see the different definitions and discussions around randomization. *(RN)* Very useful to hear patient transcripts for reflection on own current process and response to patients *(S3)* Good to have discussions with how other trials have overcome recruitment issues *(S1)* Very useful to see how many senior clinicians struggle to recruit and get useful tips *(S2)* It was interesting to see that we all have the same difficulties in recruitment. Good to hear techniques/other nurses' approaches. *(RN)* 3. Value of discussing challenges to those who felt they were already sufficiently aware I enjoyed the day but overall I felt this course would be more beneficial to nurses new to research. However, it is always good to talk and re-evaluate my practice *(RN)* I think the study day has proved to me that as a team we are doing the right thing when explaining RCTs *(RN)* Box D: Impact of training on RCT recruitment practice Very difficult to be certain about this but I think my increased confidence and language may have helped 1 or 2 more people decide to take part. I have found those that are strongly against are still difficult to change the mind of, but either way I feel I am doing a better job of informing them of the options and explaining the benefits. *(S3)* It has made me aware of my limitations and improved my skills on how to recruit a patient *(S2)* Made me aware of the kind of language I use when discussing trials and trying to be clear with examples about things like randomization and why we use a placebo *(S3)* Workshop gave me some ideas for different approach and explanation of RCT *(RN)* Discussing clinical equipoise and how the randomization process works has been easier *(S3)* I found it easier to tackle the issues with patients who do not want to be randomized - rather than just accepting that they don't want to take part I have felt able to explore the reasons they don't want to take part and try and answer worries/concerns *(S3)*

*Abbreviation:* RCT, randomized controlled trial.

a Level of experience was collected in the baseline questionnaire only. As questionnaires were anonymous, we were not able to link participant data and state level of experience in those who provided comments after training.

#### 3.2.2 Self-confidence after training

Immediately after the workshops, participants scored high confidence levels in relation to how they might now feel discussing recruitment to RCTs with patients (in general rather than in relation to specific aspects)—mean score for surgeons 8.0 (SD, 0.9) and nurses 8.0 (SD, 1.1) (with 10 being very confident). High confidence levels persisted at follow-up after having had a chance to implement training (Table 3). There was evidence of an increase in self-confidence scores after training compared with before for every 1 of the 10 questions related to discussing various aspects of RCT recruitment—range of mean scores before training 5.1–6.9 and after 6.9–8.2, with 10 being most confident (all 95% CIs are above 0 and all *P* values <0.05) (Table 3). The greatest increases in scores were in the areas in which they felt least confident in prior to training. For surgeons, this referred to discussions with patients who are mistrustful and suspicious about RCTs (an increase of 2 mean points, 95% CI: 1.4, 2.6, *P* < 0.0001); for nurses, this was in relation to obtaining authentic informed consent for randomization from patients who have a deferential attitude toward them (an increase of 2.3 mean points, 95% CI: 1.4, 3.1, *P* < 0.0001).

Participants provided free-text comments that illuminated the increased confidence scores (Table 4, Box B). They felt the workshop improved their confidence in how to structure discussions with patients, particularly with regard to conveying equipoise and randomization. It also increased their comfort in responding to patients who decline participation to ensure that their decision is fully informed (Table 4, Box B).

### 3.3 Impact of training on raising awareness of recruitment challenges

Immediately after training, participants reported feeling highly aware of the challenges of recruitment to RCTs. The mean level of awareness reported by surgeons was 8.8 (SD, 1.2) and 8.4 (SD, 1.3) for nurses (with 10 being “very aware”). Comments supported a high level of awareness (Table 4, Box C1).

When asked at the end of the workshop how much they felt they had learnt, surgeons reported a mean score of 8.0/10 (SD, 1.3) and nurses 6.7/10 (SD, 2.3) (with 10 being highest). At up to 3 months posttraining, participants still viewed the training as useful in their discussions about RCTs with patients, with surgeons scoring higher on this than nurses—surgeons mean score 8.1/10 (SD, 1.3) and nurses mean score 7.0/10 (SD, 2.2).

Analysis of free-text comments revealed that participants specifically valued the presentations and discussions around the challenges of conveying key RCT terminology, including equipoise and randomization, as well as the discussions around terminology, balancing treatment arms and addressing patients' treatment preferences. Having “real” examples from actual RCTs was deemed helpful. Attendees further valued the interactive nature of the workshop, enabling them to share recruitment difficulties and learn from the experiences of others (Table 4, Box C2).

A sizeable proportion of nurses had been recruiting to RCTs for a number of years, and several had received similar training from the QuinteT team previously but in a more condensed and trial-specific format. Some nurses therefore felt that the course was “preaching to the converted” and that it would be better suited to those with less experience and training, thereby explaining their lower scores on how much they considered they had learnt. They did, however, appreciate the importance of re-evaluating their practice, and it offered confirmation that they were doing the “right thing” (Table 4, Box C3).

### 3.4 Impact of training on RCT recruitment practice

Immediately after the workshop participants felt the training was likely to make at least some difference to their future discussions of RCTs with patients, with surgeons feeling this difference to be greater than nurses—mean score 8.1 (SD, 1.3) for surgeons and 6.6 (SD, 2.6) for nurses (with 10 being “a lot of difference”). This was demonstrated in practice as at up to 3 months follow-up half of the surgeons reported that the training had made “a lot of difference” to their RCT recruitment discussions with patients (19/38, 50%). Slightly fewer nurses reported this (10/25, 40%). Only 2/38 surgeons and 2/25 nurses reported the training as making “no difference”. Participants felt that the workshop had made them aware of their training needs and improved their recruiting skills, offering ideas for different approaches and explanations. Specific reference was made to improvements in discussions with regard to conveying equipoise, explaining randomization, and exploring treatment preferences (Table 4, Box D).

## 4 Discussion

In this study, we developed training that was tailored to support doctors' and research nurses' identified needs in recruiting patients to challenging RCTs, for example, those with diverse treatment options. The aim of the training was to increase self-confidence in discussing RCTs with potential participants and improve self-assessed recruitment practice. Content focused on raising awareness and encouraging discussion of clear obstacles and hidden emotional and intellectual challenges to recruitment. Following the QuinteT RCT Recruitment Training, surgeons and nurses reported increased self-confidence in discussing RCTs with patients, a raised awareness of hidden challenges and improvements in how they felt they conveyed key RCT concepts and responded to treatment preferences. This study, therefore, supports the use of training workshops for recruiters to equip and educate them in dealing with recruitment into RCTs in surgery. It has implications for the development and implementation of future training for RCT recruiters.

Despite the literature highlighting training needs for health professionals in recruiting patients to RCTs [8], [12], [13], [14], [31], there have been relatively few initiatives to address this. Prior interventions have tended to focus on issues around interaction and communication with patients and structuring RCT discussions in line with agreed ethical practice [14]. Recruitment difficulties are often perceived to arise from practical, logistical, and patient-related issues but may also reflect recruiters' underlying discomforts associated with their dual clinical and research roles [8], [25], [27], [32]. This discomfort is evident among recruiters demonstrating concern with “bothering” patients [25], [27], [32] and unease with going against their clinical judgment in recruiting them to an RCT [26]. It is also evident in delivering interventions [33] and closing down an RCT [34]. These emotional challenges can result in eligible patients not being approached or being steered toward a particular treatment without the opportunity to fully discuss and consider RCT participation. Raising recruiters' awareness of these issues, while demonstrating the impact they can (often unknowingly) have on recruitment and offering suggestions to minimize them, forms the unique basis of the QuinteT RCT Recruitment Training.

A comparison of recruiter training programs to identify what aspects of training are most effective at optimizing recruitment is hampered by variation in the content, delivery, and format of training, in addition to varying measures of effectiveness [14]. A recent systematic review identified 17 studies of varying quality that evaluated training programs for RCT recruiters [14]. Training mostly included a mix of health professionals covering general aspects of RCTs (such as core principles of RCTs and discussing key concepts with patients) as well as trial-specific issues. Training programs increased recruiter confidence in communicating key information to patients in early [17] and later phased cancer RCTs [18], [19], [20]. In these studies, recruiter-perceived confidence was measured before and after training using a tool that was adapted for the present study. Our findings of increased recruiter confidence following training confirm those from these studies.

Participants in the present study most valued training on conveying equipoise, explaining randomization and exploring treatment preferences—aspects that were attributed to the increased confidence scores and impact on practice. We know from previous research that recruiters can struggle with explaining randomization in a way that patients readily understand [35], [36], [37]. Communicating clinical equipoise has also been shown to be particularly challenging for recruiters [12], [25], [26], [38]. Despite intentions to set aside personal treatment biases and to present treatments in a neutral and balanced way, recruiters can override or compromise clinical equipoise in patient encounters [38]. Patients' decisions around RCT participation can be guided by treatment biases conveyed (often subtly) by the recruiting clinician [39], [40], [41], [42]. This emphasizes the importance of encouraging recruiters to reflect on personal biases and raise awareness of how and in what ways these can be transmitted to patients at a cost to recruitment. Prejudiced views of recruiters on trial interventions have been cited as a key reason for recruitment failure in discontinued RCTs [8]. Patient treatment preferences pose a further difficulty for recruiting staff. There is an assumption that preferences act as a barrier to recruitment [43] and that “challenging” them borders on coercion [44]. However, exploring treatment preferences can be justified on the basis of gauging patients' level of understanding of the study treatments to ensure that their decision whether to participate or not is well-informed. Recruiters can be trained to elicit and address preferences during RCT recruitment consultations, and this can lead to an increase in the numbers of patients who then consider and accept participation [28], [29], [36], [45]. Workshop attendees openly expressed how they were now more comfortable exploring patients' treatment preferences rather than accepting an initial preference at face value.

The impact of training in terms of self-confidence in recruiting patients to RCTs appeared greater on the whole for nurses than surgeons, although the study was not powered to detect differences across the different health professionals. This finding may have been because surgeons had less opportunity than nurses to put this training into practice in the 3-month follow-up period. Implementing new skills and adapting existing ones are likely to require practice. As nurses had more RCT experience at the outset than surgeons, it is also possible that training reaffirmed nurses' confidence in recruiting. Some nurses highlighted this posttraining. A further observation was that surgeons' baseline self-confidence scores regarding patients who are most critical and suspicious of research were relatively low before training. This was repaired to some extent by the training. A key aspect of the training was managing patients' treatment preferences, which can arise from being suspicious of RCTs [28]. It seems that the training may have been tapping into a previously unmet need. However, both observations require further exploration in a future, larger evaluation powered to detect differences across groups and in recruitment rates.

A key feature of our training intervention is the separate training of doctors and nurses. Previous research has demonstrated that support and training needs for doctors and nurses overlap, but doctors need support to ensure they are committed to the RCT design and comfortable with the eligibility criteria and interventions options, whereas nurses need support in relation to perceived role conflicts and training on approaching eligible patients [25]. Nurses have taken on an increasing role in RCT recruitment in recent years, but research has highlighted a perceived conflict between their roles in caring for patients and recruiting them to an RCT [25]. This conflict has resulted in patients missing the opportunity to consider participation. Similarly, doctors have shown difficulty in reconciling their research with clinical practice roles, admitting that when faced with a particular patient or those with specific clinical characteristics, they become more uncomfortable in inviting them on the RCT [26]. Surgery is a particular branch of medical science in which the conduct of RCTs is often considered too challenging to undertake for practical and cultural reasons [46]. Surgeons often have less exposure to conducting RCTs than other clinical colleagues (e.g., oncologists). As a result, they tend to have less familiarity with and understanding of this methodology [2] and seem to struggle more with setting aside their personal treatment biases and recruiting patients to RCTs [26]. The findings in the present study of enhanced self-confidence and perceived impact on practice following training of surgeons become even more salient when considered in this context. These issues justify training health professionals separately to enable focus on their specific training needs, but recognizing the value that training different health professionals together can also bring [47].

Our workshop materials were designed with reference to health care professional training initiatives delivered by other groups [14] and developed from empirical research that has identified recruitment challenges [25], [26] and techniques that appear to have been effective in improving recruitment through QRIs [24]. The training is essentially a behavior change intervention defined as a “coordinated set of activities designed to change specified behavior patterns” [48]—in this case health professionals' RCT recruitment practice. The behavior change techniques that were addressed in the intervention include “feedback and monitoring,” “shaping knowledge,” “natural consequences,” and “comparison of behavior” within the behavior change technique taxonomy by Michie et al [49]. The general attributes of our training are supported by Michie et al.'s behavior change wheel (BCW) which helps to select the intervention function most likely to be effective in changing a particular target behavior [48]. Based on our prior empirical work [24], [25], [26], targeting the “psychological capability” and “reflective motivation” determinants of behavior had the greatest potential to result in behavior change. According to the BCW, the most appropriate interventions for addressing these determinants are “training” and “education,” which fits with our type of intervention. Furthermore, based on a theory-led overview of published systematic reviews of professional behavior change interventions, educational meetings and “educational outreach visits” (where a skilled trainer/facilitator imparts knowledge) are more likely to successfully change professional behavior than other types of interventions [50]. It will be important to further consider our training intervention in the context of theory, with a view to identifying the active ingredients to make it more effective.

The main limitation of the study is that we were unable to assess the impact of training on actual practice in terms of recruiting patients and relied instead on self-assessed impact. While this may not be an objective reflection of impact, participants were consistent in their responses—perceiving immediately after training that it was likely to have some impact on how they discussed RCTs with patients and then reporting it as having had an impact at up to 3-month follow-up. Moreover, free-text responses were similar from the different health professionals and highlighted the key training areas—consideration of conveying equipoise, explaining randomization, and exploring treatment preferences. Although response rates at 1- to 3-month follow-up were high (72% for surgeons and 81% for nurses), it is possible that those who did not reply did not experience an impact of training on their recruitment practice or did not recruit patients in this time period. We acknowledge that there may be a degree of selection bias in terms of the types of people who agreed to the training, but personal invites from the chief investigator or trial manager resulted in each RCT having their key recruiters attend. As questionnaires from the majority of attendees were anonymous, we were unable to account for paired responses, which if analyzed using an appropriate regression model might have increased the reliability of the findings. It also meant that we were unable to undertake stratified analysis or explore a group effect. With regard to the latter point, the direction of change of self-confidence scores after training compared with before is consistent for both surgeons and nurses and mirrors the findings from other studies [17], [18], [19].

A further limitation is that we developed and evaluated our own training intervention. Findings are preliminary, as training was delivered in the relatively early stages of development, but they are promising and in line with other similar studies [17], [18], [19]. The intervention would need more robust testing by others when it is fully developed, with a clinically relevant effect specified and sample size calculation. Most of the participants invited to the training workshops were recruiting to RCTs that the trainers were coapplicants on or were aware of as having/likely to have recruitment difficulties (due, for example, to diverse treatments). Some of them had already had exposure to similar training from the QuinteT team within their individual RCT. This may have diluted findings so it may be possible that the observed effects could have been greater if the recruiters had not had prior exposure to similar training material. The strength of this work is the focus on raising awareness of issues which are often hidden to recruiters and yet can unknowingly impact on recruitment. Using evidence from real RCTs, we were able to not only highlight the key issues that similar recruiting health professionals have, but also demonstrate the impact of this on the patients' decision-making process around recruitment to RCTs. The full impact from this kind of awareness and self-reflection may take longer to be seen in practice than the follow-up period in this study. This suggests that impact may still be observed further on in time, although continued training may be required to ensure effects do not dissipate over time.

The training provided support to those working on a number of diverse RCTs within the field of surgery, as opposed to focusing on the issues inherent within a particular trial. Working within individual RCTs to identify and address specific issues may lead to a greater impact. The QRI, a two-staged process implemented in RCTs deemed difficult for recruitment or those experiencing difficulties, explores where the key difficulties in the trial might be focused. Strategies are then employed to address them (largely involving feedback and training akin to that given within the present study) [24]. There seems to be a place for both broad RCT recruitment training covering common issues and training within an individual trial covering trial-specific issues that could together enhance informed consent and recruitment in RCTs.

## 5 Conclusions

A 1-day training workshop for health professionals that focuses on the emotional and intellectual challenges of recruiting patients to RCTs looks promising in increasing confidence with recruitment, raising awareness of hidden challenges and impacting positively on self-assessed recruitment practice. Although the training intervention evaluated in this study was in the relatively early stages of development, findings are encouraging. The intervention will continue to be developed, made more available, and be evaluated in relation to informed consent and recruitment rates.
