# Supplementary material for: Training health professionals to recruit into challenging randomized controlled trials improved confidence: the development of the QuinteT randomized controlled trial recruitment training intervention
Source: J Clin Epidemiol. 2018 Mar;95:34–44. doi: 10.1016/j.jclinepi.2017.11.015 (PMC5844671; doi:10.1016/j.jclinepi.2017.11.015)
Supplement: Appendix 1 [file mmc1.docx]

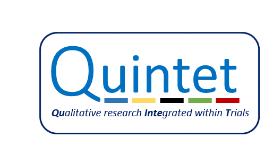

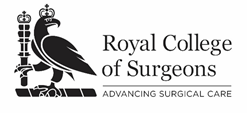

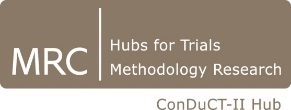

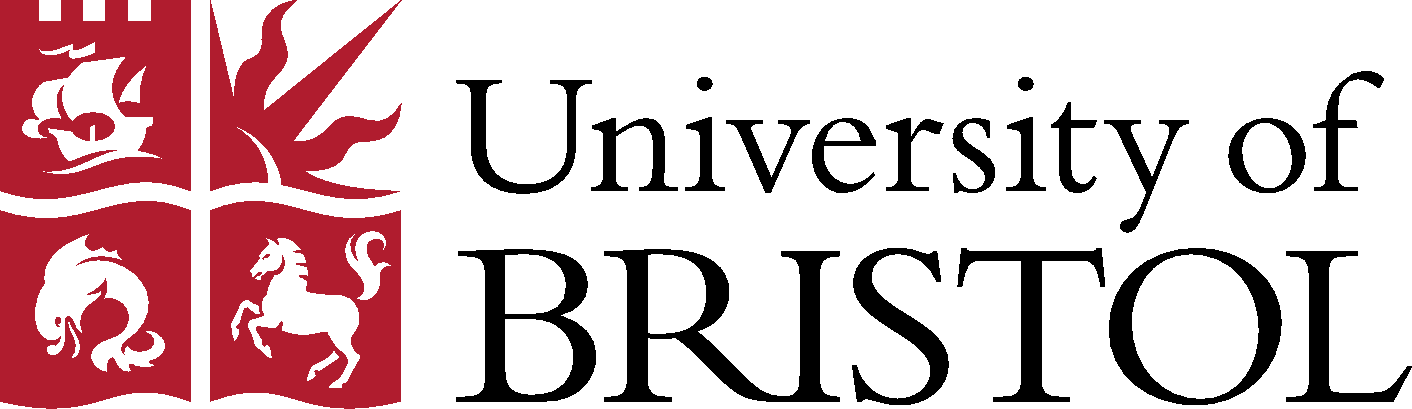


**Optimising Recruitment into RCTs in surgery**

***(Various dates in 2015 and 2016)***

**School of Social and Community Medicine, University of Bristol, Canynge Hall, 39 Whatley Road, Bristol BS8 2PS, Room LG.08**

10.30-11.00 Registration with tea and coffee

11.00-11.10 Welcome

*(Professor Jane Blazeby)*

11.10-11.30 Why the need for randomised trials?

(*Dr Sara Brookes)*

11.30-12.30 Initial challenges recruiting to surgical RCTs

*(Professor Jane Blazeby)*

12.30-12.50 Patient treatment preferences

(*Dr Nicola Mills)*

12.50-1.35 *Lunch*

1.35-2.20 Hidden challenges to RCT recruitment (I)

*(Professor Jenny Donovan)*

2.20-2.40 *Tea and Coffee break*

2.40-3.10 Hidden challenges to RCT recruitment (II)

*(Dr Leila Rooshenas)*

3.10-3.45 Conveying RCT terminology
*(Dr Marcus Jepson)*

3.45-4.00 Close
 *(Professor Jenny Donovan and Dr Nicola Mills)*
