# Supplementary material for: Training health professionals to recruit into challenging randomized controlled trials improved confidence: the development of the QuinteT randomized controlled trial recruitment training intervention
Source: J Clin Epidemiol. 2018 Mar;95:34–44. doi: 10.1016/j.jclinepi.2017.11.015 (PMC5844671; doi:10.1016/j.jclinepi.2017.11.015)
Supplement: Appendix 3 [file mmc3.docx]

In conjunction with MRC HTMR ConDuCT-II Hub and the University of Bristol

**Optimising recruitment into RCTs in surgery**

**A focused workshop for research nurses**

20^th^ April 2015

**Key points for ‘difficult’* trials**

**Making recruitment work**

- Acknowledge that recruitment is complex and difficult, and different to clinical practice
- Integrate the study into your clinic
- Use screening logs to help assess patient eligibility
- Understand the level of equipoise (your own and that of the team)
- Convey the study with enthusiasm and the treatments/ options with care and balance
- Ask patients to “keep an open mind”
- If patients seem to have a preference, gently explore why and ensure they are informed
- Explain randomisation as a method to achieve a fair comparison between excellent options
- Explain the next steps before the patient leaves
