# Supplementary material for: Training health professionals to recruit into challenging randomized controlled trials improved confidence: the development of the QuinteT randomized controlled trial recruitment training intervention
Source: J Clin Epidemiol. 2018 Mar;95:34–44. doi: 10.1016/j.jclinepi.2017.11.015 (PMC5844671; doi:10.1016/j.jclinepi.2017.11.015)
Supplement: Appendix 4 [file mmc4.docx]

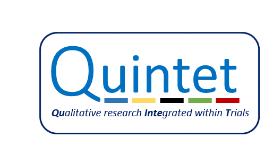

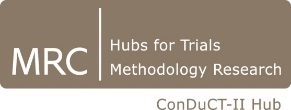

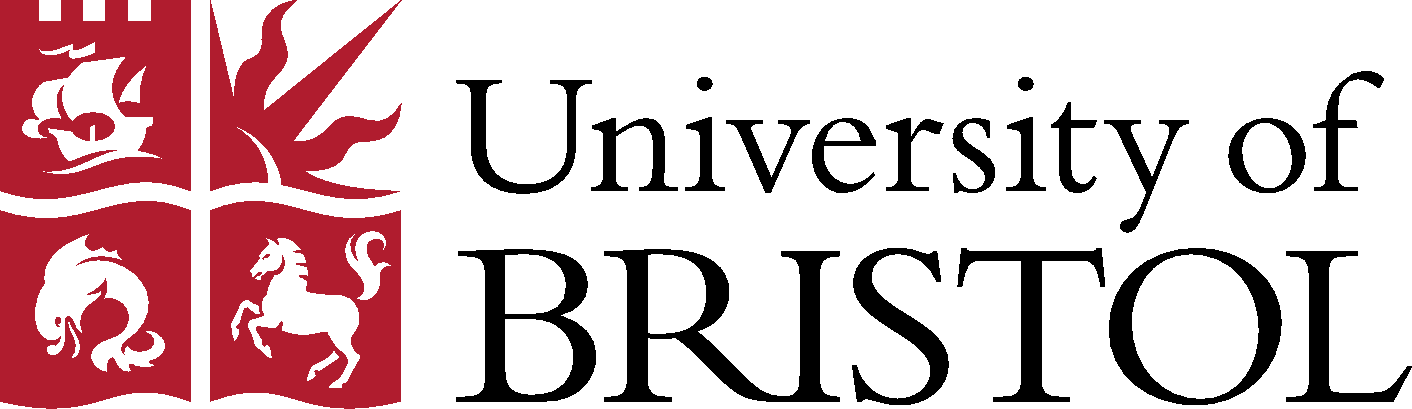


**Optimising Recruitment into RCTs**

**Key points**

Recruitment to RCTs is complex, can be difficult, and is different to clinical practice. This document, developed by the Quintet team at the University of Bristol, includes evidence-based suggestions that can help with recruitment and informed consent to RCTs.

**Approaching patients**

- Approach all eligible patients so that everyone has a chance to consider participation
- Present the study enthusiastically
- Explain the benefits of study participation, e.g. close follow-up and monitoring, and that the aim of research is to produce evidence so that future patients will not have to face current treatment uncertainties
- Explain that patients have the right to decide whether or not to take part, and it will not affect their treatment in any way
- Integrate the study into clinical practice

**Discussing the study**

- Introduce the study early on, and avoid the term ‘trial’
- Ask patients to ‘keep an open mind’ until you have given them all the information
- Explain uncertainty as the rationale for the study
- Consider your own level of equipoise and if you are unwittingly conveying treatment biases
- Present balanced information about all treatment options
  - Avoid loaded terminology (i.e. gold standard, experimental, first treatment)
  - Balance the advantages and disadvantages of each treatment
- If a patient has a preference, gently ask them why so you can uncover any misunderstandings and ensure they make an informed decision

**Describing randomisation**

- It is important to explain (a) the rationale for randomisation (to achieve a fair comparison between treatments) and (b) that they have an equal chance of having any of the treatments
- It is helpful to avoid using terms such as ‘toss of a coin ‘or ‘decided by a computer’ when explaining randomisation
- Check they would consider accepting any of the treatments before arranging randomisation

May 2016
